# The Gender-Specific Interaction of *DVL3* and *GSK3β* Polymorphisms on Major Depressive Disorder Susceptibility in a Chinese Han Population: A Case-Control Study

**DOI:** 10.1155/2022/2633127

**Published:** 2022-01-28

**Authors:** Siyuan Ke, Jiarui Li, Lu Zhao, Jiarun Yang, Xueyan Zhao, Wenxin Zhang, Xiaohui Qiu, Xiuxian Yang, Jiawei Zhou, Yuying Tong, Xiongzhao Zhu, Xuan Liu, Yanjie Yang, Zhengxue Qiao, Tianyi Bu

**Affiliations:** ^1^Department of Medical Psychology of Harbin Medical University, Heilongjiang Province, China 150081; ^2^Department of Medical Education Management, School of Health Management of Harbin Medical University, Harbin, China 150081; ^3^Department of Psychology School of Education of Heilongjiang University, Heilongjiang Province, China 150080; ^4^Medical Psychological Institute of the Second Xiangya Hospital of Central South University, Hunan Province, China 410011

## Abstract

Based on the “oxidative stress hypothesis” of major depressive disorder (MDD), cells regulate their structure through the Wnt pathway. Little is known regarding the interactions of dishevelled 3 (*DVL3*) and glycogen synthase kinase 3 beta (*GSK3β*) polymorphisms with MDD. The aim of the current study was to verify the relationship between *DVL3* and *GSK3β* genetic variants in a Chinese Han population and further to evaluate whether these interactions exhibit gender-specificity. A total of 1136 participants, consisting of 541 MDD patients and 595 healthy subjects, were recruited. Five single-nucleotide polymorphisms (SNPs) of *DVL3*/*GSK3β* were selected to assess their interaction by use of a generalized multifactor dimensionality reduction method. The genotype and haplotype frequencies of *DVL3*/*GSK3β* polymorphisms were significantly different between patients and controls for *DVL3* rs1709642 (*P* < 0.01) and *GSK3β* rs334558, rs6438552, and rs2199503 (*P* < 0.01). In addition, our results also showed that there were significant interaction effects between *DVL3* and *GSK3β* polymorphisms and the risk of developing MDD, particularly in women. The interaction between *DVL3* (rs1709642) and *GSK3β* (rs334558, rs6438552) showed a cross-validation (CV) consistency of 10/10, a *P* value of 0.001, and a testing accuracy of 59.22%, which was considered as the best generalized multifactor dimensionality reduction (GMDR) model. This study reveals the interaction between *DVL3* and *GSK3β* polymorphisms on MDD susceptibility in a female Chinese Han population. The effect of gender should be taken into account in future studies that seek to explore the genetic predisposition to MDD relative to the *DVL3* and *GSK3β* genes.

## 1. Introduction

Major depressive disorder (MDD) is a common mental disorder, afflicting more than 300 million people globally [1]. Highlighting how depressive disorders have become the leading cause of years lived with disability worldwide, nearly 8 million patients suffering from MDD die annually as a result of suicide [2]. However, as matters stand, the diagnostic capacity and efficacy of predicting the risk for developing MDD is still suboptimal. This is mostly because the pathophysiology of MDD is not entirely clear. To better understand the genetic etiology of MDD, great effort has been made to identify candidate genes. Recent evidence shows that MDD is associated with alterations in brain function, neuronal plasticity, and reduced volume of the frontal cortex and the hippocampus [3]. Preclinical and clinical studies have shown that increased generation of ROS (reactive oxygen species, ROS) and exhaustion of antioxidative defences are responsible for the altered brain structure. This hypothesis is known as the “oxidative stress hypothesis” of MDD [4].

Stress can have a physiological effect in the form of oxidative stress. In order to cope with this stress, cells adapt their constitution via several pathways including the Wnt pathway [5]. Brain samples of patients suffering from major depressive disorder are found to exhibit a dysregulation of Wnt signalling activity. Changes in Wnt signaling components were also observed in mouse models of depression. Disheveled (*DVL*) is one of the downregulated genes in the nucleus accumbens (NAc) of mice susceptible to social failure stress [6]. Downregulation of *DVL* increases *GSK3β* activity, and the overexpression of *GSK3β* induces depression-like behavior [7]. Therefore, the Wnt/DVL/GSK3*β* signaling cascade may modulate MDD susceptibility.

Recently, two large, comprehensive genomic and transcriptomic studies have implicated *DVL3* as a gene associated with a significant risk of developing MDD. Determined by the Psychiatric Genomics Consortium, the *DVL3* polymorphism rs1969253 was found to have the strongest association with MDD in individuals with European ancestry [8]. Although this mega-analysis of genome-wide association study (GWAS) included 9240 MDD cases and 9519 controls, genome-wide significance was not observed (*P* = 4.8 × 10^−6^). Nevertheless, in a subsequent gene expression study by Jansen et al. which included 882 MDD, 635 remitted MDD, and 331 control individuals, it was observed that *DVL3* gene expression was upregulated in MDD cases [9]. This provided further evidence suggesting that the *DVL3* gene has a role to play in MDD. Taken together, findings from both studies strongly suggest that the *DVL3* gene may play an important role in the development of MDD in European populations. However, the association between *DVL3* gene and MDD was still not clear in the populations of non-European ancestry.

*DVL3* codes for the segment polarity protein disheveled (*DVL*), which plays a critical role in dendritic arborization [10]. Previous studies suggested that the effect of *DVL* on MDD is mainly mediated via glycogen synthase kinase 3 beta (*GSK3β*). Using depression models, Wilkinson et al. found that the downregulation or blocking of *DVL* promoted depression-like behavior and that this effect may be mediated via *GSK3β* [11]. Sutton et al. suggested that antipsychotics alleviate psychosis as they might indirectly act on *DVL* to initiate downstream *GSK3β* changes [12]. Thus, it can be seen that *GSK3β* is a crucial mediator in the effect of *DVL* on MDD, and that they have an actual biological interaction, that is, that the activation of *DVL* can inhibit *GSK3β* activity via phosphorylation.

*GSK3β* is a serine/threonine protein kinase that plays a role in regulating neuronal plasticity and cell survival [13]. Substantial evidence has been provided for the involvement of *GSK3β* in the pathophysiology of MDD. Firstly, inhibition of *GSK3β* by administering antidepressants promotes axonal formation and elongation in mature neurons [14, 15] and maintains hippocampal cell turnover and synaptic plasticity [16]. Specific *GSK3β* inhibitors were also found having antidepressant-like effect [17, 18]. Moreover, the change in *GSK3β* activity has not only proven to be associated with MDD but has also shown to be associated with the suicidal behavior of depressive patients, depressive symptoms, and the severity of MDD [19, 20]. In addition, *GSK3β* polymorphisms were also reported to be associated with MDD. The rs334558 single-nucleotide polymorphism (SNP) was shown to be associated with an increased risk for developing MDD in an Asian population from a recent meta-analysis, including 2,311 case and 2,535 control individuals [21]. This SNP was associated with the promoter region of *GSK3β* which influences transcriptional strength [22] and protein activity [14]. Moreover, a *GSK3β* haplotype block which includes the rs6438552 and rs334558 polymorphisms was also found to interact with both the gray matter volume in the right hippocampus and superior temporal gyri [23] and the disease status regions of the brain, such as left thalamus, in MDD cases [24]. However, the association between *GSK3β* SNPs and MDD was also not replicated in the subgroup of 3231 patients with recurrent depression and 3186 controls among Chinese Han women [25].

Based on these observations, the *DVL3* and *GSK3β* genes appear to play a significant role in the genesis of MDD, but results are inconsistent. Previous studies have found that the interaction among gene-gene weakens the association between a single gene and MDD [26, 27]. Thus, this study investigated the relationship of the *DVL3* and *GSK3β* genes to MDD by evaluating the effect that their interaction has on MDD susceptibility in a Chinese Han population. About 45% of the genetic susceptibility to MDD is not shared between genders [25]. It has been suggested that the association between the *DVL3*/*GSK3β* genes and MDD is different between men and women [8, 21]. Thus, this study also sought to validate the sex-specific effect of *DVL3* and *GSK3β* genes on MDD susceptibility. To our knowledge, this study is the first to report the association of *DVL3* polymorphisms and their sex-specific effect on MDD by way of gene-gene interaction analysis in a Chinese Han population. Resultant data will contribute to elucidating the underlying genetic architecture of *DVL3* in Chinese Han MDD patients as well as provide precious etiological clues for explaining the pathogenesis of MDD.

## 2. Material and Methods

### 2.1. Participants

A total of 1136 participants, which consisted of 541 first-episode MDD patients and 595 age-matched healthy subjects, were recruited for this study between February 2014 and December 2016 at a single hospital. All participants were of Chinese Han origin and were living in the same geographical area in the north of China. Cases were diagnosed with MDD in a single psychiatric hospital according to the Fourth Edition of the Diagnostic and Statistical Manual of Mental Disorders (DSM-IV). Each patient was interviewed independently by at least two psychiatrists using the Structured Clinical Interview for DSM-IV disorders and was evaluated using the 24-item Hamilton Rating Scale for Depression. Only patients with a minimum HAMD score of 21 were included in this study. Not a single patient received any antidepressant treatment within 4 weeks preceding assessment. Cases with a history of brain organic mental disorders, a family history of genetic disorders, intellectual disability, or those who recently received blood transfusion treatment were excluded from the study. Healthy subjects with a family history of mental disorders were also excluded from participating.

The study was approved by the Harbin Medical University Research Ethics Committee, China. Written informed consent forms were obtained from all participants in our study.

### 2.2. DNA Isolation and Genotyping

Genomic DNA was isolated from collected EDTA-anticoagulated blood samples using the AxyPrepTM Blood Genomic DNA Minprep Kit (Axygen, Union City, CA, USA). SNPs in same haplotype blocks, which have been reported as having strong epidemiological credibility for involvement with MDD (*DVL3*: rs1969253, rs1709642; *GSK3β*: rs334558, and rs6438552, rs2199503) were selected. Primers for polymerase chain reaction (PCR) amplification were designed with Primer 5.0 software, and final specific primers were checked using NCBI-BLAST. Probe sequences and descriptions for SNPs are displayed in Table 1. The ABI PRISM 7900 Sequence Detection System (Applied Biosystems, Foster City, CA, USA) and ABI 3730 DNA sequencer (Applied Biosystems) were used for genotyping and purifying.

### 2.3. Statistical Analysis

Using SPSS 21.0 software, Student's *t*-test was used to compare the means of continuous variables between cases and controls, and the differences in the distribution of categorical variables were evaluated using the chi-square test. Bonferroni correction was applied to the *P* value to correct for multiple testing. Haploview software (version 4.2) was used to perform Hardy–Weinberg equilibrium tests and pairwise linkage disequilibrium and haplotype association analyses, and a permutation test with 1000 replications was used to measure empirical *P* values. Gene–gene interactions were analyzed using generalized multifactor dimensionality reduction (GMDR) software (version 0.9). The best gene-gene interaction model based on the values arising from cross-validation (CV) consistency and accuracy testing was selected. A permutation test with 1000 replications was used to measure empirical *P* values thereby substantiating the significance of the model. A corrected *P* value<0.05 (two-tailed) was considered to be statistically significant.

## 3. Results

### 3.1. Preliminary Analyses

Basic characteristics of participants are displayed in Table 2. The mean age of cases and controls was 43.99 years versus 42.91 years, respectively. In present study, there were 171 males (31.6%) and 370 females (68.4%) in MDD group and 214 males (36.0%) and 381 females (64.0%) in control group. There was no significant statistical difference between cases and controls in gender, age, and marital status distribution (*P*_gender_ = 0.109, *P*_age_ = 0.121, *P*_marital_ = 0.487). HAMD score of MDD group was in the range of 21~58 points, and mean score was 31.25 points.

### 3.2. Single-Marker Association with MDD

The potential associations between MDD and the studied polymorphisms were examined (Table 3). For *DVL3* polymorphisms, significant differences in genotypic and allelic distributions between cases and controls were confirmed at locus rs1709642 (*P* < 0.01 for both genotype and allele) and rs1969253 (*P* = 0.007 for genotype and *P* = 0.021 for allele); but after Bonferroni correction, the difference in allelic distributions at locus rs1969253 was not significant. Odds ratio analysis results showed that T-allele carriers of rs1709642 were more likely to suffer from MDD than C-allele carriers (OR = 1.36, 95% CI: 1.15-1.60). In total sample, the significantly genotypic and allelic distribution differences between cases and controls were confirmed at all *GSK3β* SNPs; after Bonferroni correction, the differences were still significant. Odds ratio analysis results showed that A-allele carriers of rs334558 and rs6438552 were more likely to suffer from MDD than G-allele carriers (rs334558: OR = 1.41, 95% CI: 1.19-1.67; rs6438552: OR = 1.45, 95% CI: 1.23-1.71). And G-allele carriers of rs2199503 were more likely to suffer from MDD than A-allele carriers (OR = 1.36, 95% CI: 1.14-1.61).

### 3.3. Association Analysis Based on Different Genetic Model

And we examined the potential associations between MDD and the studied polymorphisms on different genetic models (Table 4). The dominant model of *DVL3*/*GSK3β* polymorphisms showed the better fit for association. Odds ratio analysis results showed that homozygous mutant or heterozygote carriers for the five SNPs were more likely to suffer from MDD than those with wild-type alleles after adjustment for gender, age, smoking, and alcohol drinking.

### 3.4. Haplotype Association with MDD

Pairwise linkage disequilibrium analysis between *DVL3*/*GSK3β* SNPs was measured in this population. Strong linkage disequilibrium was observed between rs1969253 and rs1709642, as well as rs6438552, rs2199503, and rs334558. The *D*′ value between rs1969253 and rs1709642 was 0.966, 0.975 between rs6438552 and rs2199503, 0.875 between rs6438552 and rs334558, and 0.827 between rs2199503 and rs334558. As all gene markers were in strong linkage disequilibrium (Table 5), we further tested haplotype effects. The results of the haplotype analysis are shown in Table 6. Statistically significant differences were observed in three haplotype distributions after permutation test were performed (*DVL3* rs1709642 and rs1969253: C-C; *GSK3β* rs334558, rs6438552, rs2199503: G-G-A and A-A-G). The occurrence of the *DVL3* C-C haplotype and *GSK3β* G-G-A haplotype was observed significant less in case than in control individuals. The occurrence of *GSK3β* A-A-G haplotype was observed significant more in case than in control individuals.

### 3.5. Gene-Gene Interaction Analysis with GMDR

The gene–gene interaction effects in MDD susceptibility were detected using a GMDR model with age, gender, smoking, and alcohol consumption as covariates. Results of the GMDR analysis are summarized in Table 7. The *P* value was determined using the permutation test with 1000 replications. Significant two-locus to five-locus interaction models were observed. As results suggested that there was a significant interaction between the *DVL3* (rs1709642, rs1969253) and *GSK3β* (rs334558, rs6438552, and rs2199503) polymorphisms on MDD susceptibility (*P* < 0.05), the interaction between *DVL3* (rs1709642) and *GSK3β* (rs334558, rs6438552) showed a CV consistency of 10/10 and a testing accuracy of 55.31% which was considered as the best multilocus model.

### 3.6. Association Analysis after Gender Stratification

We stratified this total sample according to gender. After stratification by gender, we detected the relationship of *DVL3*/*GSK3β*polymorphisms with MDD by evaluating their single and interaction effects upon MDD susceptibility. In female sample, the significantly genotypic and allelic distribution differences between cases and controls were confirmed at locus rs334558, rs6438552, and rs1709642, and the distribution differences of rs2199503 were observed only in allele (Table 8). In male sample, we did not find association between the MDD and *DVL3*/*GSK3β* polymorphisms. In haplotype analysis, the results from the overall sample were also replicated only in the female sample (Table 9). Gene-gene interactions on MDD susceptibility were detected using a GMDR model which incorporated age, smoking, and alcohol consumption as covariates. Results are summarized in Table 10. The *P* value was determined using the permutation test with 1000 replications. In female, significant model of two-locus and three-locus gene-gene interaction with MDD was observed (*P* < 0.05). And an interaction between *DVL3* (rs1709642) and *GSK3β* (rs334558, rs6438552) was considered as the best multilocus model with a CV consistency of 10/10 and a testing accuracy of 59.22%. The results from the overall sample were replicated only in the female sample with a higher testing accuracy of 59.22%. However, in male samples, no significant differences were observations in the interactions among five SNPs and their impact on MDD (*P* > 0.05). Relative to the *DVL3* and *GSK3β* polymorphisms, this result suggests that there might be a significant effect on MDD susceptibility in female, but not male patients.

## 4. Discussion

In this study, we investigated the relationship between *DVL3*/*GSK3β* polymorphisms and MDD risk in a Chinese Han population. This study also sought to validate the effects that the gene-gene interaction has on MDD susceptibility. We found that a 1.36-fold increase in allele frequency was observed in those who carried a T-allele at *DVL3* gene SNP site rs1709642. Those in whom these SNPs were identified were statistically more likely to suffer from MDD. For *GSK3β* polymorphisms, individuals with an A-allele had a higher risk of developing MDD than those who carried a G-allele at *GSK3β* gene SNP site rs334558/rs6438552, and individuals with a G-allele had a higher risk of developing MDD than those who carried an A-allele at *GSK3β* gene SNP site rs2199503. Our results also showed that a dominant model of inheritance was a better fit for the association between MDD and *DVL3*/*GSK3β* polymorphisms. In haplotype analysis, the individuals with a statistically reduced risk of developing MDD were found to have haplotypes containing the rs1969253-C and rs1709642-C alleles. In *GSK3β* haplotypes, the individuals with a G-G-A haplotype (rs334558, rs6438552, and rs2199503) were found to have a statistically reduced risk of developing MDD, while individuals carrying an A-A-G haplotype were at a greater risk of developing MDD. In addition, our result showed that there were significant interaction effects between *DVL3* and *GSK3β* polymorphisms on the risk of developing MDD. It is observed that a three-locus interaction model among *DVL3* SNP rs1709642 and *GSK3β* SNPs rs334558 and rs6438552, which was shown to be highly consistent and have a maximum accuracy of 55.31%, was the best multilocus model that could be constructed.

Although research on *DVL3* role in mental disorders has just started, it has been found that its mRNA expression levels is significantly decreased in the nucleus accumbens and frontal regions of those patients who are depressed [9, 11]. For the *GSK3β* gene, a prior study found that rs334558 and rs6438552 are associated with MDD endophenotypes, which include symptoms of anxiety and P300 latency. Individuals with an A-allele in rs334558 and rs6438552, respectively, have a longer P300 latency and higher amplitude [28]. Previous observations have also found that the rs6438552 SNP is associated with variations in gray matter volume in the right hippocampus and bilateral superior temporal gyri in MDD cases [23]. When comparing MDD cases with a rs6438552 G/G genotype to those with an A-allele at the same position, A-allele carriers showed increased nodal centralities in the limbic system, thalamus, and parts of the parietal, temporal, occipital, and frontal regions [24]. In addition, and prior to Bonferroni correction, rs2199503 has been association with MDD in a Chinese Han cohort of 1,045 MDD, and 1,235 control individuals [29], which is consistent with our results. Inkster et al., however, did not find a correlation between rs2199503 SNP and gray matter volume in a study on the correlation between the *GSK3β* gene polymorphism and brain structure in patients with MDD [23]. For that reason, our study warrants replication in future studies.

Intriguingly, we found that there might be differences in the effect of the *DVL3*/*GSK3β* gene interaction on the MDD susceptibility based on gender. After stratification according to sex, it was observed that the interactions between *DVL3* and *GSK3β* polymorphisms on MDD susceptibility were significant only in female cases. This observation was repeated by evaluating SNP and haplotype effects on MDD susceptibility in our sample. The testing accuracy of the three-locus model was also higher in female cases (59.22%). Relative to *DVL3*/*GSK3β* polymorphisms, this finding indicated that gender might have a specific effect on the pathogenesis of MDD in a Chinese Han population. A separate study found that *DVL3* rs1969253 was potential associated with MDD in female, but not in male samples [8]. Similarly, such an association was observed in our study before Bonferroni correction. In addition, we found that rs1709642 was also possible susceptibility loci for MDD in female. A recent meta-analysis, which was successfully replicated in a Chinese Han sample, validated that there is a significant association between rs334558 and MDD in female Asian patients [21]. Lin et al. have also reported an association between rs6438552 and female patients with an older age of onset of bipolar I disorder [30]. These findings strongly supported the notion that gender may modify the effects that the *DVL3*/*GSK3β* genes have on MDD susceptibility. The inconsistent results of the association of *GSK3β* polymorphisms on MDD may therefore have arisen due to sample differences in sex ratios (F/M: 0.43-1.28 in negative studies, 2.16 in our total sample).

It has been reported that the higher prevalence of MDD in women compared to men may be due to sex hormones [31]. There is evidence that *DVL* and *GSK3β* may be involved in the pathogenesis of MDD via Wnt or NF-*κ*B signaling [32, 33]. Both of these signaling pathways are known to be regulated by estrogen [34, 35]. This might explain why *DVL3* and *GSK3β* genes played a more significant role in female MDD cases.

However, these associations were not replicated in the CONVERGE Consortium study that included Chinese Han women with recurrent depressive disorder and a high rate of melancholic symptoms. We suspect that the inconsistency in the results may have arisen due to sample differences including the number of MDD attacks, the proportion of melancholic MDD, and different stages of MDD, for example. In addition, it is unclear that whether immune response, neuroplasticity, or other factors contributed to the failure to replicate genetic effects of *DVL3* gene in the CONVERGE sample [36–41].

Interpretation of these results should consider the following limitations. Firstly, the geographic and ethnic origin was strictly controlled in this study in order to reduce the potential effects of population stratification. Our results are therefore difficult to extrapolate to other populations, which creates challenges in that results need to be further verified in other ethnicities and regions. Secondly, our selected SNPs are located in the same haploblock and may not represent the genetic information in other regions of the *DVL3*/*GSK3β* gene. Thirdly, the sample size was relatively small. It is necessary to verify these results in a large MDD population.

## 5. Conclusion

To our knowledge, this study is the first to reveal the sex-specific interaction of *DVL3* and *GSK3β* genes on MDD susceptibility in a Chinese Han population. The effect of gender should be taken into account in future studies that seek to explore the genetic predisposition to MDD relative to the *DVL3* and *GSK3β* genes. This study is also the first to reveal the underlying genetic architecture of *DVL3*, including genetic loci frequencies, effect sizes, models of action, and interaction with other loci, in a Chinese Han population. These factors are important determinants for the success in identifying genetic associations for disease(s) with complex traits. Although their specific mechanism still needs further study, the results of our study suggest that interactions between the *DVL3* and *GSK3β* genes are involved in the pathogenesis of MDD in female Chinese Han patients.

## Figures and Tables

**Table 1:** Primers sequence for SNPs of *DVL3* and *GSK3β.*

Gene	SNP ID	Polymorphisms	Location	Primer sequence (5′→3′)	MAF
*DVL3*	rs1709642	C/T	Intron region	F: 5′-TATCGAGGGAGAAAAGACAGG-3′ R: 5′-GTAACGTTTCCTATGCCGTTC-3′	0.4429
	rs1969253	C/A	Intron region	F: 5′-GAAAGTGGGTCAGAGGAGGA-3′ R:5′-TGGCTGGGCCTACCCTGAGCTTG-3′	0.4804

*GSK3β*	rs334558	G/A	5′regulatory region	F: 5′-TCAGGAAGTGTCCGCGCTTTG-3′ R: 5′-AAGGAGGTGGAGGACGAGTAG-3′	0.4014
	rs6438552	G/A	Intron region	F: 5′-TCTAAACCTTAAAGAACTTAG-3′ R: 5′-TCTTTTTTGCAGAGCAAGGTG-3′	0.4065
	rs2199503	A/G	Intron region	F: 5′-GGCAGGTTGGAATTTCAGAAC-3′ R: 5′-CTGGAGGTGAATCTTAGTGAG-3′	0.2021

**Table 2:** Demographic characteristics of participants.

Variables	Case (*n* = 541)	Control (*n* = 595)	*χ* ^2^/*t*	*P*
Age (years)	43.99 ± 12.92	42.91 ± 9.14	-1.606	0.109
Gender *N* (%)			2.402	0.121
Female	370 (68.4)	381 (64.0)		
Male	171 (31.6)	214 (36.0)		
Marital status *N* (%)			1.438	0.487
Single	83 (15.3)	77 (12.9)		
Stable	423 (78.2)	481 (80.8)		
Separated or widow	35 (6.5)	37 (6.3)		

The genotypic distributions of all selected *DVL3* and *GSK3β* polymorphisms conformed to the Hardy–Weinberg equilibrium (*P* > 0.05).

**Table 3:** Analysis on the association between *DVL3/GSK3β* SNPs and MDD risk.

SNP	Sample	Genotype *N* (%)			*P*	Allele *N* (%)		*P*	OR (95% CI)
*DVL3*									
rs1709642		CC	CT	TT		C	T		
	Case	93 (17.2)	311 (57.5)	137 (25.3)	**<0.01**	497 (45.9)	585 (54.1)	**<0.01**	1.36 (1.15-1.60)
	Control	174 (29.2)	289 (48.6)	132 (22.2)		637 (53.5)	553 (46.5)		
rs1969253		CC	CA	AA		C	A		
	Case	107 (19.8)	301 (55.6)	133 (24.6)	**0.035**	515 (47.6)	567 (52.4)	0.105	1.21 (1.03-1.43)
	Control	165 (27.7)	294 (49.4)	136 (22.9)		624 (52.4)	566 (47.6)		
*GSK3β*									
rs334558		GG	GA	AA		G	A		
	Case	152 (28.1)	281 (51.9)	108 (20.0)	**<0.01**	585 (54.1)	497 (45.9)	**<0.01**	1.41 (1.19-1.67)
	Control	233 (39.2)	277 (46.5)	85 (14.3)		743 (62.4)	447 (37.6)		
rs6438552		GG	GA	AA		G	A		
	Case	124 (22.9)	290 (53.6)	127 (23.5)	**<0.01**	538 (49.7)	544 (50.3)	**<0.01**	1.45 (1.23-1.71)
	Control	214 (36.0)	273 (45.9)	108 (18.1)		701 (58.9)	489 (41.1)		
rs2199503		AA	AG	GG		A	G		
	Case	185 (34.2)	261 (48.2)	95 (17.6)	**0.010**	631 (58.3)	451 (41.7)	**<0.01**	1.36 (1.14-1.61)
	Control	259 (43.5)	261 (43.9)	75 (12.6)		779 (65.5)	411 (34.5)		

Significant results are provided in bold after Bonferroni correction.

**Table 4:** Analysis on the association between *DVL3/GSK3β* SNPs and MDD risk on different genetic models.

Gene	SNP	Dominant model		Recessive model	
		OR (95% CI)	*P*	OR (95% CI)	*P*
*DVL3*	rs1709642	2.09 (1.56-2.81)	**<0.01**	1.19 (0.90-1.59)	0.220
	rs1969253	1.61 (1.21-2.15)	**0.005**	1.09 (0.82-1.45)	0.541

*GSK3β*	rs334558	1.72 (1.33-2.23)	**<0.01**	1.42 (1.03-1.95)	0.175
	rs6438552	1.94 (1.48-2.55)	**<0.01**	1.36 (1.01-1.83)	0.215
	rs2199503	1.49 (1.16-1.91)	**0.010**	1.47 (1.04-2.06)	0.140

Dominant: AA versus Aa + aa; recessive: AA + Aa versus aa (a is the minor allele). Significant results are provided in bold after Bonferroni correction. Adjustment for gender, age, smoking, and alcohol drinking.

**Table 5:** The result of linkage disequilibrium analysis between *GSK3β* and *DVL3* SNPs.

SNP1-SNP2	LOD	*D*′	*r* ^2^
rs6438552-rs2199503	282.19	0.975	0.696
rs6438552-rs334558	241.91	0.875	0.653
rs2199503-rs334558	207.97	0.826	0.587
rs1709642-rs1969253	417.28	0.966	0.925

**Table 6:** Haplotype-based association analysis results.

Haplotypes	SNP	Case ratios	Control ratios	*χ* ^2^	*P* ^∗^
*DVL3*	rs1709642-rs1969253				
H1	C-C	0.456	0.523	10.199	**0.005**
H2	T-A	0.520	0.463	7.444	0.051

*GSK3β*	rs334558-rs6438552-rs2199503				
H1	G-G-A	0.472	0.557	16.421	**<0.01**
H2	A-A-G	0.373	0.301	13.316	**0.001**
H3	A-A-A	0.061	0.043	3.823	0.291
H4	G-A-G	0.039	0.038	0.022	1.000
H5	G-A-A	0.029	0.029	0.001	1.000
H6	A-G-A	0.021	0.026	0.535	0.992

**Table 7:** The gene-gene interaction models obtained by GMDR.

Locus no.	Best model	Testing accuracy (%)	CV consistency	*P*
2	*DVL3*(rs1709642), *GSK3β* (rs6438552)	52.43	7/10	0.377
**3**	***DVL3*(rs1709642), *GSK3β* (rs334558, rs6438552)**	**55.31**	**10/10**	**0.001**
4	*DVL3*(rs1709642), *GSK3β* (rs334558, rs6438552, rs2199503)	54.36	10/10	0.011
5	*DVL3*(rs1709642, rs1969253), *GSK3β* (rs334558, rs6438552, rs2199503)	53.47	10/10	0.011

For SNP: 0=no risk alleles; 1=1 risk allele; 2=2 risk alleles; adjustment for age, gender, smoking, and alcohol drinking; *P* value was yielded by the sign test; empirical *P* value was obtained using the permutation test (1000 replication).

**Table 8:** Association between *DVL3/GSK3β* SNPs and MDD in different gender subgroups.

SNP	Genotype/allele	Female			Male		
		Case (*n* = 370)	Control (*n* = 381)	*P*	Case (*n* = 171)	Control (*n* = 214)	*P*
*DVL3*							
rs1969253	CC	75 (20.3)	113 (29.7)	0.050	32 (18.7)	52 (24.3)	0.405
	CA	206 (55.7)	181 (47.5)		95 (55.6)	113 (52.8)	
	AA	89 (24.0)	87 (22.8)		44 (25.7)	49 (22.9)	
	C	356 (48.1)	407 (53.4)	0.200	159 (46.5)	217 (50.7)	0.246
	A	384 (51.9)	355 (46.6)		183 (53.5)	211 (49.3)	
rs1709642	CC	64 (17.3)	119 (31.2)	**<0.01**	29 (17.0)	55 (25.7)	0.117
	CT	214 (57.8)	179 (47.0)		97 (56.7)	110 (51.4)	
	TT	92 (24.9)	83 (21.8)		45 (26.3)	49 (22.9)	
	C	342 (46.2)	417 (54.7)	**0.001**	155 (45.3)	220 (51.4)	0.094
	T	398 (53.8)	345 (45.3)		187 (54.7)	208 (48.6)	
*GSK3β*							
rs334558	GG	101 (27.3)	151 (39.6)	**0.005**	51 (29.8)	82 (38.3)	0.212
	GA	190 (51.3)	175 (45.9)		91 (53.2)	102 (47.7)	
	AA	79 (21.4)	55 (14.5)		29 (17.0)	30 (14.0)	
	G	392 (53.0)	477 (62.6)	**<0.01**	193 (56.4)	266 (62.1)	0.132
	A	348 (47.0)	285 (37.4)		149 (43.6)	162 (37.9)	
rs6438552	GG	83 (22.4)	137 (36.0)	**<0.01**	41 (24.0)	77 (36.0)	0.155
	GA	193 (52.2)	176 (46.2)		97 (56.7)	97 (45.3)	
	AA	94 (25.4)	68 (17.8)		33 (19.3)	40 (18.7)	
	G	359 (48.5)	450 (59.1)	**<0.01**	179 (52.3)	251 (58.6)	0.080
	A	381 (51.5)	312 (40.9)		163 (47.7)	177 (41.4)	
rs2199503	AA	124 (33.5)	165 (43.3)	0.065	61 (35.7)	94 (43.9)	0.208
	AG	176 (47.6)	164 (43.1)		85 (49.7)	97 (45.4)	
	GG	70 (18.9)	52 (13.6)		25 (14.6)	23 (10.7)	
	A	424 (57.3)	494 (64.8)	**0.015**	207 (60.5)	285 (66.6)	0.082
	G	316 (42.7)	268 (35.2)		135 (39.5)	143 (33.4)	

**Table 9:** Analysis of association between *DVL3/GSK3β* haplotype and MDD in different gender subgroups.

Haplotype	Female		*χ* ^2^	*P*	Male		*χ* ^2^	*P*
	Case	Control			Case	Control		
*DVL3*								
C-C	0.459	0.533	8.083	**0.031**	0.447	0.505	1.581	0.606
T-A	0.516	0.451	6.304	0.095	0.529	0.484	2.502	0.814
*GSK3β*								
G-G-A	0.459	0.557	14.599	**<0.01**	0.501	0.557	2.385	0.619
A-A-G	0.385	0.301	11.655	**0.002**	0.349	0.301	1.995	0.729
A-A-A	0.059	0.039	3.056	0.563	0.065	0.048	0.983	0.946
G-A-G	0.039	0.043	0.189	1.000	0.040	0.028	0.780	0.966

Note: *DVL3*: rs1709642-rs1969253; *GSK3β*: rs334558-rs6438552-rs2199503.

**Table 10:** The gene-gene interaction models obtained by GMDR after gender stratification.

Samples	Locus no.	Best model	Testing accuracy (%)	CV consistency	*P*
Female	2	*DVL3*(rs1709642), *GSK3β* (rs6438552)	59.19	10/10	0.011
	**3**	***DVL3*(rs1709642), *GSK3β* (rs334558, rs6438552)**	**59.22**	**10/10**	**0.001**
	4	*DVL3*(rs1709642), *GSK3β*(rs334558, rs6438552, rs2199503)	56.58	10/10	0.055
	5	*DVL3*(rs1709642, rs1969253), *GSK3β*(rs334558, rs6438552, rs2199503)	55.42	10/10	0.055
Male	2	*DVL3*(rs1709642), *GSK3β* (rs2199503)	51.17	5/10	0.377
	3	*DVL3*(rs1709642), *GSK3β*(rs6438552, rs2199503)	58.04	10/10	0.055
	4	*DVL3*(rs1709642), *GSK3β*(rs334558, rs6438552, rs2199503)	54.12	8/10	0.377
	5	*DVL3*(rs1709642, rs1969253), *GSK3β*(rs334558, rs6438552, rs2199503)	54.27	10/10	0.377

For SNP: 0=no risk alleles; 1=one risk allele; 2=two risk alleles. Adjustment for age, smoking, and alcohol drinking. Empirical *P* value was obtained using the permutation test (1000 replication).

## Data Availability

The datasets used or analysed during the current study are available from the corresponding author on reasonable request.

## Abbreviations

CV: Cross-validation
DSM-IV: Diagnostic and Statistical Manual of Mental Disorders, Fourth Edition
DVL: Dishevelled
GMDR: Generalized multifactor dimensionality reduction
GSK3*β*: Glycogen synthase kinase 3 beta
GWAS: Genome-wide association study
HAMD: Hamilton Rating Scale for Depression
LD: Linkage disequilibrium
MDD: Major depressive disorder
PCR: Polymerase chain reaction
SNP: Single-nucleotide polymorphism.
